# Supplementary material for: The CfSnt2-Dependent Deacetylation of Histone H3 Mediates Autophagy and Pathogenicity of Colletotrichum fructicola
Source: J Fungi (Basel). 2022 Sep 18;8(9):974. doi: 10.3390/jof8090974 (PMC9506038; doi:10.3390/jof8090974)
Supplement: Supplementary file 1 [file jof-08-00974-s001.zip › jof-1846455-supplementary.pdf]

Table S1. The expression levels of *CfATG* genes in transcriptome data

Gene ID	Gene name	Δ*Cfgcn5*/WT	P value
gene-CGGC5_v001583	*CfATG1*	1.265	0.016
gene-CGGC5_v006476	*CfATG2*	1.532	9.47E-07
gene-CGGC5_v011295	*CfATG3*	2.480	1.09E-19
gene-CGGC5_v013845	*CfATG4*	1.794	3.84E-10
gene-CGGC5_v006481	*CfATG5*	1.325	0.037
gene-CGGC5_v012401	*CfATG6*	1.243	0.025
gene-CGGC5_v015799	*CfATG7*	2.115	1.72E-08
gene-CGGC5_v010022	*CfATG8*	2.908	4E-27
gene-CGGC5_v013854	*CfATG9*	1.378	0.001
gene-CGGC5_v001121	*CfATG10*	1.937	8.07E-08
gene-CGGC5_v014758	*CfATG12*	4.288	1.01E-24
gene-CGGC5_v014851	*CfATG13*	4.284	1.94E-66
gene-CGGC5_v007470	*CfATG15*	1.126	0.323
gene-CGGC5_v001805	*CfATG16*	1.092	0.445
gene-CGGC5_v008931	*CfATG18*	1.345	0.001

Table S2 Primers used in this study

Primer name	Sequence (5’-3’)	Remark
qRT1F	TGATGGTATTTGCGAAGAGG	qRT-PCR Primer of v012815
qRT1R	TGTAGGTTCCGACGAAGATTAG	qRT-PCR Primer of v012815
qRT2F	ATCATCGTCGTCGCCATCC	qRT-PCR Primer of v008601
qRT2R	GCTTGCCAGTCTTGAGGTTGT	qRT-PCR Primer of v008601
qRT3F	AGAAGGCGTGGAACTCGTA	qRT-PCR Primer of v004552
qRT3R	CGTGTTTGGTAGAGGGGAG	qRT-PCR Primer of v004552
qRT4F	CCGCTCTACAACGCCACTAA	qRT-PCR Primer of v001103
qRT4R	AACTTGCCCGTCGCCATAC	qRT-PCR Primer of v001103
qRT5F	TTTGGCAGGTCAGGTTGGG	qRT-PCR Primer of v004561
qRT5R	GCTGGATGGGGAGCGGTAT	qRT-PCR Primer of v004561
qRT6F	TGGTCCAAGACGGCATTGAG	qRT-PCR Primer of v014933
qRT6R	GGTGGGAGCGACGAAGAGA	qRT-PCR Primer of v014933
qRT*CfATG1*F	AAACTGGGGACGGACTTGG	qRT-PCR Primer of *CfATG1*
qRT*CfATG1*R	ATCCTGAGCCGCCTTTTCT	qRT-PCR Primer of *CfATG1*
qRT*CfATG2*F	CAAGAACTCCCTCGAAACAACA	qRT-PCR Primer of *CfATG2*
qRT*CfATG2*R	GCTCAATCCGCCAAACCAG	qRT-PCR Primer of *CfATG2*
qRT*CfATG3*F	GAGGATTTCCCCTTCTTCGC	qRT-PCR Primer of *CfATG3*
qRT*CfATG3*R	CTGGTCGTCAACCTCGCTTT	qRT-PCR Primer of *CfATG3*
qRT*CfATG4*F	TGGGTCGGTCATACAATCT	qRT-PCR Primer of *CfATG4*
qRT*CfATG4*R	TCCATCTTGAACAGCGTAATC	qRT-PCR Primer of *CfATG4*
qRT*CfATG5*F	CCCGACGCTGTTCCCATCCA	qRT-PCR Primer of *CfATG5*
qRT*CfATG5*R	CGACCGTGACACAGAGCCAACC	qRT-PCR Primer of *CfATG5*
qRT*CfATG6*F	CACTCCTCACGAATCCCACA	qRT-PCR Primer of *CfATG6*
qRT*CfATG6*R	CCTTTTGAACCAGCCTTGC	qRT-PCR Primer of *CfATG6*
qRT*CfATG7*F	TCGGCTTGGTTCCTCACAC	qRT-PCR Primer of *CfATG7*
qRT*CfATG7*R	CAGCACCCTCCTCTTCATCTT	qRT-PCR Primer of *CfATG7*
qRT*CfATG8*F	CCGATCTCCCATCATCCCG	qRT-PCR Primer of *CfATG8*
qRT*CfATG8*R	TGCTCCTCGTAGATGCTGCTC	qRT-PCR Primer of *CfATG8*
qRT*CfATG9*F	ATTTTGGTCTTTGGCTGTATGT	qRT-PCR Primer of *CfATG9*
qRT*CfATG9*R	CTCTTTGGATTGGTTCCGTAT	qRT-PCR Primer of *CfATG9*
qRT*CfATG10*F	TGCCACCACTTTGACAGCC	qRT-PCR Primer of *CfATG10*
qRT*CfATG10*R	TTCAGCATCTATCATAGCCTCGT	qRT-PCR Primer of *CfATG10*

qRT*CfATG12*F	TAAGTTCAAGCCCGTGGG	qRT-PCR Primer of *CfATG12*
qRT*CfATG12*R	GCCAAAGGCTGGGTTCATAG	qRT-PCR Primer of *CfATG12*
qRT*CfATG13*F	AAAACGATACCCACCAACG	qRT-PCR Primer of *CfATG13*
qRT*CfATG13*R	ATACAGCGGATGCCGAAGC	qRT-PCR Primer of *CfATG13*
qRT*CfATG15*F	GGGATGTTTGGACGAGACG	qRT-PCR Primer of *CfATG15*
qRT*CfATG15*R	GAGGTGATGAGATGCGAAGTG	qRT-PCR Primer of *CfATG15*
qRT*CfATG16*F	AACTCTCCGACCGCATCGC	qRT-PCR Primer of *CfATG16*
qRT*CfATG16*R	ATCTGCTTCGTGTTCTCCCTGT	qRT-PCR Primer of *CfATG16*
qRT*CfATG18*F	ACAGTGACGGAAATGTGGGA	qRT-PCR Primer of *CfATG18*
qRT*CfATG18*R	AACCGAGAGCTGCTTGACC	qRT-PCR Primer of *CfATG18*
qRT*ACTIN*F	ATCAACCCCAAGTCCAACAG	qRT-PCR Primer of *ACTIN*
qRT*ACTIN*R	CGATTTCACGCTCGGCAGT	qRT-PCR Primer of *ACTIN*
